# Left Ventricular Geometry and Inferior Vena Cava Diameter Co-Modify the Risk of Cardiovascular Outcomes in Chronic Hemodialysis Patients

**DOI:** 10.3390/medicina60071140

**Published:** 2024-07-15

**Authors:** Chung-Kuan Wu, Ming Wang, Zih-Kai Kao, Noi Yar, Ming-Tsang Chuang, Tzu-Hao Chang

**Affiliations:** 1 Division of Nephrology, Department of Internal Medicine, Shin-Kong Wu Ho-Su Memorial Hospital, Taipei 111, Taiwan; chungkuan.wu@gmail.com (C.-K.W.); ql4t86ql4t86@gmail.com (M.W.); yar.noi.1392@gmail.com (N.Y.); 2 School of Medicine, Fu-Jen Catholic University, New Taipei 242, Taiwan; 3 Institute of Biophotonics, National Yang Ming Chiao Tung University, Taipei 112, Taiwan; zkkao@ym.edu.tw; 4 Clinical Data Center, Office of Data Science, Taipei Medical University, Taipei 11031, Taiwan; mtchuang@tmu.edu.tw; 5 Graduate Institute of Biomedical Informatics, Taipei Medical University, Taipei 11031, Taiwan; 6 Clinical Big Data Research Center, Taipei Medical University, Taipei 11031, Taiwan

**Keywords:** concentric left ventricular hypertrophy, eccentric left ventricular hypertrophy, inferior vena cava diameter, major cardiovascular event, mortality, hemodialysis

## Abstract

*Background and Objectives*: Left ventricular hypertrophy (LVH) represents a significant cardiovascular risk in patients undergoing chronic hemodialysis (CHD). A large inferior vena cava diameter (IVCD), potentially indicative of fluid overload and a contributing factor to elevated cardiovascular risk, has not been sufficiently explored. Therefore, our study aims to gain further insights into this aspect. *Materials and Methods*: A retrospective cohort study enrolled patients receiving CHD in a single medical center with available echocardiography from October to December 2018. They were categorized into four groups based on LVH geometry and IVCD. Cox proportional hazard models assessed the risk of major adverse cardiovascular effects (MACEs) and cardiovascular and overall mortality after multivariate adjustments. Kaplan–Meier analysis depicted MACE-free events and survival during the follow-up time. *Results*: Of the 175 CHD patients, 38, 42, 45, and 50 exhibited small IVCD with eccentric and concentric LVH and large IVCD with eccentric and concentric LVH, respectively. Compared to small IVCD and eccentric LVH, large IVCD and eccentric LVH had the highest risk of MACEs, followed by large IVCD and concentric LVH (aHR: 4.40, 3.60; 95% CI: 1.58–12.23, 1.28–10.12, respectively). As for cardiovascular mortality, large IVCD and concentric LVH had the highest risk, followed by large IVCD and eccentric LVH, and small IVCD and concentric LVH. (aHR: 14.34, 10.23, 8.87; 95% CI: 1.99–103.35, 1.41–74.33; 1.01–77.87). The trend in overall mortality risk among the groups was similar to that of cardiovascular mortality. *Conclusions*: LVH geometry and IVCD co-modify the risk of MACEs and cardiovascular and overall mortality in CHD patients. The highest risk of MACEs is associated with large IVCD and eccentric LVH, while the highest risk of cardiovascular and overall mortality is linked with large IVCD and concentric LVH.

## 1. Introduction

Chronic kidney disease (CKD), affecting more than 10% of the population worldwide [1], is a leading cause of morbidity. Cardiovascular disease (CVD) is a major cause of morbidity and mortality in patients with end-stage kidney disease (ESKD) [2]. More than half of ESKD patients on dialysis have CVD, which increases their risk of mortality by 20 times compared to the general population [3]. This heightened risk of CVD is attributed to traditional risk factors including hypertension, diabetes mellitus, dyslipidemia, smoking, and aging, along with CKD-related risk factors such as hyperphosphatemia, secondary hyperparathyroidism, uremic toxins, inflammation and oxidative stress, and left ventricular hypertrophy [4]. Left ventricular hypertrophy (LVH), a form of CVD [5], is linked to coronary heart disease, heart failure, arrhythmias, sudden cardiac death, stroke, and all-cause mortality [6], particularly in those on dialysis. Systemic arterial resistance, volume-related hypertension, anemia, and oxidative stress are common pathophysiologic risk factors of ESKD patients with LVH [7]. With the prevalence of LVH being 75% at the time of dialysis initiation, individuals with LVH had poor cardiovascular outcomes and a high mortality risk [2,7].

LVH is a condition characterized by an increase in the left ventricular (LV) mass due to wall thickening, enlargement of the LV chamber, or both [8]. Chronic pressure overload causes an abnormal increase in LV myocardial mass, resulting in concentric LVH, whereas volume overload is the mechanism underlying LV dilation and compensatory eccentric LVH [9]. In ESKD patients, both volume and pressure overload are common. The diameter of the inferior vena cava (IVCD) may be influenced by total blood volume and heart function, which are related to LVH geometry [10].

The IVCD and its response to respiration were often used to assess right atrial (RA) pressure [11]. Additionally, IVCD and collapsibility can be used to monitor intravascular volume or central venous pressure [12]. Optimal fluid volume control in chronic hemodialysis (CHD) patients is vital as excessive volume increases the risk for cardiovascular complications and mortality [13]. Therefore, a large IVCD is associated with poor prognosis, including mortality [14]. Cardiovascular mortality, however, has been less addressed. Furthermore, the effects of LVH geometry modified by IVCD on cardiovascular events and mortality remain unknown.

## 2. Materials and Methods

### 2.1. Study Population

**Figure 1:**
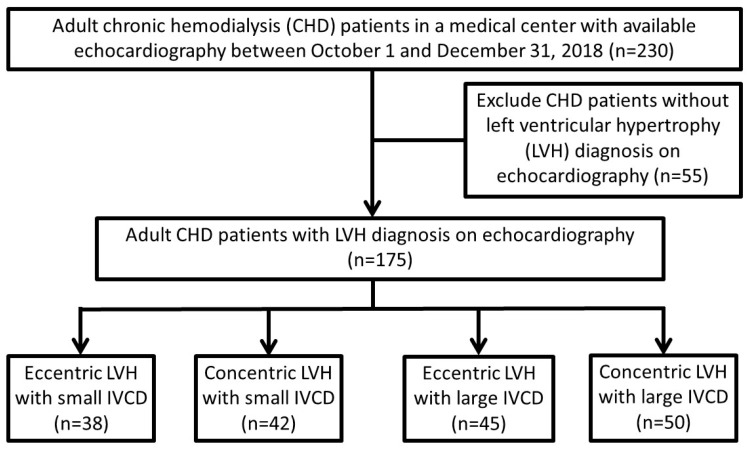
Flowchart of the study for chronic hemodialysis (CHD) patients based on left ventricular hypertrophy (LVH) geometry and inferior vena cava diameter (IVCD).

A total of 230 ESKD patients receiving hemodialysis for more than 3 months in a single medical center, with available echocardiographic results between 1 October and 31 December 2018, were enrolled. Fifty-five CHD patients without LVH were excluded. The patients were categorized into four groups based on LVH geometry (eccentric or concentric) and IVCD severity (large or small), as shown in Figure 1. The cutoff point for IVCD, 1.5 cm, was determined using receiver operating curve analysis to minimize the difference between the sensitivity and specificity based on our previous study [15].

### 2.2. Collections of Comorbid, Dialysis, and Medical History and Laboratory Data

The patients’ demographic and comorbid data, dialysis clearance efficacy, laboratory parameters, and concurrent medications were collected initially. Baseline echocardiographic parameters including left ventricular end-diastolic diameter (LVEDD), left ventricular end-systolic diameter (LVESD), left ventricular posterior wall (LVPW) thickness, left atrium diameter, interventricular septum (IVS) thickness, and IVCD were documented. The enrolled patients were followed up until the occurrence of mortality or 31 December 2020, whichever came first.

### 2.3. Measurement of IVCD and LVH

Standardized transthoracic echocardiography was performed by experienced cardiologists during the non-dialysis mid-week day. A transducer was placed in the subxiphoid region to obtain long- and short-axis views of the inferior vena cava (IVC). The IVCD was measured during maximal inspiration and expiration to avoid Valsalva-like effects on the M-mode electrocardiogram. Values were obtained in the parasternal long-axis view and at or immediately below the tips of the mitral valve leaflets, in accordance with the guidelines of American Society of Echocardiography and European Association of Cardiovascular Imaging Chamber Quantification [16]. The two-dimensional-echocardiography-guided M-mode approach was used for the LV measurements. Concentric LVH was defined as an elevated LV mass index (LVMI) (>95 or 115 g/m^2^ in females or males, respectively) and a relative wall thickness (RWT) ≥ 0.42, while eccentric LVH was defined as an elevated LVMI with an RWT < 0.42.

### 2.4. Outcome Definition

The primary outcome was major adverse cardiovascular events (MACEs), while the secondary outcomes were cardiovascular and overall mortality. MACEs included any of the following events: hospitalization for myocardial infarction, coronary revascularization, stroke, heart failure, or death from cardiovascular causes. Cardiovascular mortality was defined as death attributable to myocardial infarction, heart failure, cardiac arrhythmia, sudden cardiac death, aortic dissection, aortic aneurysm rupture, cardiac tamponade, pulmonary embolism, or ischemic or hemorrhagic cerebrovascular accident.

### 2.5. Statistical Analysis

Baseline characteristic and echocardiographic findings of the four groups of CHD patients are described as percentage for categorical data and as mean ± standard deviation (SD) for continuous data. The chi-squared test or Fisher’s exact test was used to compare categorical variables, while one-way analysis of variance or the Kruskal–Wallis test was used to compare continuous variables with normal and skewed distribution, respectively. For MACE-free events, cardiovascular, and overall survival during follow-up, survival curves were obtained via competing risk model of Kaplan–Meier method and compared between the groups by log-rank test. Univariate Cox regression was used to estimate the relative risk (crude hazard ratio [cHR]) of MACEs, cardiovascular, and overall mortality. Significant variables, including age, sex, history of coronary artery disease, peripheral artery disease, serum total cholesterol, and serum platelet were adjusted in the multivariate Cox proportional hazard models to estimate adjusted hazard ratios (aHRs). All statistical analyses were performed using SPSS version 28 (IBM, Armonk, NY, USA) or SAS version 9.4. A two-sided *p* < 0.05 indicated significance.

## 3. Results

### 3.1. Baseline Characteristics of Study Participants

Among the 175 enrolled patients, 38 (21.7%) exhibited eccentric LVH with a small IVCD, 42 (24%) exhibited concentric LVH with a small IVCD, 45 (25.7%) exhibited eccentric LVH with a large IVCD, and 50 (28.6%) exhibited concentric LVH with a large IVCD, shown in Figure 1 and Table 1. The individuals with a small IVCD were significantly older than those with a large IVCD irrespective of LVH geometry (*p* = 0.008). Women had a smaller IVCD than men in both the eccentric and concentric LVH groups (*p* = 0.049). Coronary artery disease was significantly more prevalent in CHD patients with a large IVCD (*p* = 0.036), and peripheral artery disease (PAD) was significantly more prevalent in the concentric LVH groups (*p* = 0.011). The patients with a small IVCD had a higher level of platelet count and serum cholesterol than those with a large IVCD (*p* = 0.021 and 0.005, respectively). Regarding medications, only insulin and analogs were found to be used significantly more in the group with eccentric LVH (*p* < 0.022). 

According to the echocardiographic findings shown in Table 2, the individuals with concentric LVH had a considerably larger IVS, LVPW, and RWT than those with eccentric LVH (*p* < 0.001). The left atrium diameter and LVMI were greater in the groups with a large IVCD than in those with a small IVCD (all *p* < 0.05). The LVEDD and LVESD were significantly greater in the eccentric LVH groups than in the concentric LVH groups (*p* < 0.001).

### 3.2. Association of LVH Geometry and IVCD on MACEs

**Figure 2:**
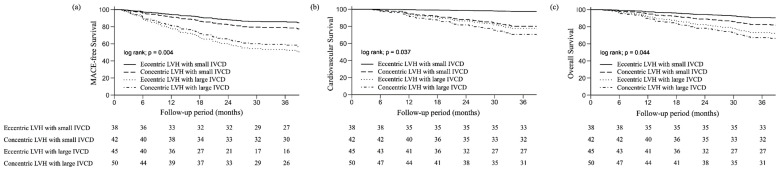
Event-free and survival curves of chronic hemodialysis (CHD) patients according to left ventricular hypertrophy (LVH) geometry and inferior vena cava diameter (IVCD). (**a**) Major adverse cardiovascular event (MACE)-free curves. (**b**) Cardiovascular survival. (**c**) Overall survival.

After approximately 36 months of follow-up, 60 CHD patients (34.3%) developed MACEs. The numbers were 8 (13.3%), 8 (13.3%), 23 (38.3%), and 21 (35%) in the groups of eccentric LVH with small IVCD, concentric LVH with small IVCD, eccentric LVH with large IVCD, and concentric LVH with large IVCD, respectively. CHD patients with an eccentric LVH with small IVCD had the lowest risk of MACE, followed by those with concentric LVH with small IVCD, concentric LVH with large IVCD, and eccentric LVH with large IVCD (*p* = 0.004; Figure 2a). 

In univariate Cox proportional hazard regression, CHD patients with a large IVCD with either eccentric LVH (HR: 3.13. 95% CI: 1.41–6.97) or concentric LVH (HR: 2.30, 95% CI: 1.01–5.21) had a significantly increased risk of MACEs compared with those with a small IVCD and eccentric LVH (Table 3). After adjusting for age and sex (model 1, Table 3) or for age, sex, and other significant factors listed in Table 1, including coronary artery disease, peripheral artery disease, cholesterol level, platelet count, and insulin analogs (model 2, Table 3), those with a large IVCD and eccentric LVH (aHR: 3.34, 4.40; 95% CI: 1.43–7.81, 1.58–12.23, respectively) or concentric LVH (aHR: 2.72, 3.60; 95% CI: 1.13–6.53, 1.28–10.12, respectively) still had a significantly higher risk of MACEs than did those with a small IVCD and eccentric LVH.

### 3.3. Association of LVH Geometry and IVCD on Cardiovascular and Overall Mortalities

Regarding secondary outcomes, 32 (18.3%) CHD patients died from cardiovascular causes, and 40 (22.8%) CHD patients died during the follow-up period. A total of 1 (3.1%), 8 (25.0%), 10 (31.3%), and 13 (40.6%) died from cardiovascular causes, while 5 (12.5%), 8 (20.0%), 12 (30.0%), and 15 (37.5%) died from any cause in those with eccentric LVH and small IVCD, concentric LVH and small IVCD, eccentric LVH and large IVCD, and concentric LVH and large IVCD, respectively. 

CHD patients with eccentric LVH and small IVCD had the lowest risk of cardiovascular mortality, followed by those with concentric LVH and small IVCD, eccentric LVH and large IVCD, and concentric LVH and large IVCD (*p* = 0.037; Figure 2b). The same trend risk of overall mortality from eccentric LVH and small IVCD to concentric LVH and large IVCD was noted (*p* = 0.044; Figure 2c).

Compared with CHD patients with eccentric LVH and small IVCD, those with concentric LVH and large IVCD had a significantly greater risk of cardiovascular mortality (HR: 11.56, 95% CI: 1.56–85.91), as also did those with eccentric LVH and large IVCD (HR: 10.26, 95% CI: 1.35–77.89) (Table 3). In model 1 and model 2 of Table 3 after clinical variables adjustment, the CHD patients with concentric LVH and large IVCD (aHR: 15.44, 14.34; 95% CI: 2.15–111.0, 1.99–103.35) and those with eccentric LVH and large IVCD (aHR: 10.79, 10.23; 95% CI: 1.51–77.19, 1.41–74.33) remained at higher risk of cardiovascular mortality than were the patients with eccentric LVH and small IVCD. The patients with concentric LVH and a small IVCD also exhibited a higher risk of cardiovascular mortality than did those with eccentric LVH and small IVCD (aHR: 8.34, 8.87; 95% CI: 1.05–66.29, 1.01–77.87) (model 1 and 2, Table 3).

All-cause mortality risk was significantly higher in the group with concentric LVH and a large IVCD (aHR: 3.79, 4.38, 95% CI: 1.41–10.23, 1.44–13.37) and those with eccentric LVH and a large IVCD (aHR: 2.75, 3.41, 95% CI: 1.01–7.47, 1.09–10.65) than in that with eccentric LVH and a small IVCD after adjusting for clinical variables (model 1 and 2, Table 3). 

## 4. Discussion

Our study suggested that older age, the female gender, and high cholesterol levels are associated with a smaller IVCD independent of LVH type, and a larger IVCD was found to correlate with higher rates of coronary artery disease. Peripheral artery disease (PAD) was more prevalent in the group with concentric LVH. Furthermore, our study revealed the lowest risk of MACEs, cardiovascular mortality, and overall mortality in individuals with eccentric LVH and small IVCD, followed by concentric LVH with small IVCD. The highest risk for MACEs was observed in patients with eccentric LVH and large IVCD, while those with concentric LVH and large IVCD exhibited the highest risk for cardiovascular and overall mortality. 

The higher proportion of females in the small IVCD group compared to the large IVCD is supported by the correlation between IVCD size and physical dimensions [17]. Additionally, patients in the small IVCD group were older than those in the large IVCD group, consistent with the age-related decrease in an IVCD study [18]. In our study, the small IVCD groups also had higher levels of cholesterol, possibly due to a lower proportion of statin use in this group. In contrast, the large IVCD groups showed a higher proportion of coronary artery disease, suggesting a potential link to higher rates of overhydration [19], which may increase the likelihood of turbulent blood flow, endothelial dysfunction, and ultimately, the risk of atherosclerotic cardiovascular disease [20,21]. Regarding left ventricular geometry, there was a higher PAD proportion in concentric LVH, which aligns with findings from Maimaitiaili et al. [22].

In our study, patients with eccentric LVH and large IVCD exhibited the highest risk of MACEs, followed by those with concentric LVH and large IVCD, concentric LVH and small IVCD, and lastly, the group with eccentric LVH and small IVCD. Previous literature has regarded volume overload as a risk factor for MACEs [23,24], and our own prior study also demonstrated that large IVCD, indicative of volume overload, correlated with an increased likelihood of MACE occurrence [15]. However, the effect of the left ventricular geometry on MACEs across different fluid statuses has been seldom reported. A study reported that patients with eccentric LVH had a higher risk of myocardial infarction and decompensated heart failure compared to those with concentric LVH among individuals with acute coronary syndrome. This disparity could be due to the Laplace equation, which indicates that the wall stress is directly proportional to chamber pressure and radius and inversely proportional to the thickness of the chamber wall. Consequently, eccentric LVH experiences relatively higher wall stress, resulting in an increased myocardial oxygen demand and contributing to myocardial infarction, and decompensated heart failure, which are parts of MACE composites [25]. Excess fluid exacerbates the difference in wall stress between eccentric LVH and concentric LVH; hence, eccentric LVH poses a higher risk of MACEs than concentric LVH in cases of large IVCD but not in small IVCD.

As for secondary outcomes, concentric LVH and large IVCD showed the highest cardiovascular mortality and overall mortality, followed by eccentric LVH and large IVCD, concentric LVH and small IVCD, and eccentric LVH and small IVCD. High volume status remained a risk for cardiovascular and overall mortality, which has been suggested in some studies [26,27,28]; however, it seemed that concentric LVH relative to eccentric LVH, irrespective of volume status had a higher risk for cardiovascular and overall mortality. Our previous study demonstrated that chronic hemodialysis patients with non-to-mild or moderate-to-severe aortic arch calcification with concentric LVH had a higher risk of cardiovascular and overall mortality than those with eccentric LVH [29]. In our study population, the prevalence of HTN was high. Koren et al. and Muiesan et al. reported that concentric hypertrophy had higher risks of cardiovascular and overall mortality than eccentric hypertrophy in patients with essential hypertension [30,31]. The poor prognosis of concentric LVH could be explained by several factors, such as impaired myocardial contractility, severe diastolic filling abnormalities, or increased oxygen consumption, which result in a higher risk of arrhythmias and sudden death [30,31,32,33]. Another study demonstrated that LVH with a low remodeling index, which is highly correlated with concentric LVH, had a high risk of mortality [34]. Although Zuijdewijn et al. reported no difference in cardiovascular and all-cause mortality between ESKD patients with concentric and eccentric LVH [35], the inconsistent result could be attributed to the lack of various assessments on volume status among ESKD patients, thereby impacting the outcomes.

Our study is limited by its retrospective nature, the database was extracted from a single medical center in Taiwan, which has a predominantly Han Chinese population, and the patients stayed mostly in urban areas; therefore, the results may not be generalizable. Echocardiography was performed at baseline, and no longitudinal measurements of the IVCD were recorded. More than one cardiologist performed the echocardiography examinations; thus, there might have been interobserver variability in the readings. In addition, another limitation of our study is the absence of lung ultrasound and BNP measurements, which are valuable tools for assessing hypervolemia. Future studies should consider incorporating these measurements to provide a more comprehensive evaluation of patients’ volume status. 

## 5. Conclusions

LVH geometry and IVCD influence the risk of MACEs, cardiovascular, and overall mortality in CHD patients. CHD patients with large IVCD and eccentric LVH were at the highest risk of MACEs, while those with large IVCD and concentric LVH faced the highest risk of cardiovascular and overall mortality. Evaluation of the IVCD and LVH geometry may be useful in CVD risk assessment and investigations of effective strategies for improving the prognosis of patients undergoing CHD.

## Figures and Tables

**Table 1:** Baseline characteristics of chronic HD patients according to LVH geometry and IVCD size.

	Eccentric LVH, Small IVCD (n = 38)	Concentric LVH, Small IVCD (n = 42)	Eccentric LVH, Large IVCD (n = 45)	Concentric LVH, Large IVCD (n = 50)	*p*
Age (years) *	72.7 ± 12.1	71.8 ± 12.4	66.7 ± 11.5	65.3 ± 12.0	0.008
Female (%) ^†^	20 (52.6)	25 (59.5)	14 (31.1)	22 (44.0)	0.049
Dry weight (kg) **	57.0 ± 12.3	58.7 ± 13.5	63.8 ± 15.0	60.1 ± 13.5	0.135
Vintage (years) **	6.1 ± 5.1	7.9 ± 6.5	7.1 ± 6.9	9.1 ± 8.3	0.233
Comorbidities (%)					
Type 2 DM^†^	17 (44.7)	14 (33.3)	24 (53.3)	24 (48.0)	0.290
Hypertension ^†^	27 (71.1)	36 (85.7)	38 (84.4)	40 (80.0)	0.341
Hyperlipidemia ^†^	19 (50.0)	22 (52.4)	28 (62.2)	27 (54.0)	0.690
CAD ^†^	12 (31.6)	11 (26.2)	24 (53.3)	23 (46.0)	0.036
PAD ^†^	6 (15.8)	10 (23.8)	5 (11.1)	19 (38.0)	0.011
Heart failure ^†^	6 (15.8)	10 (23.8)	10 (22.2)	12 (24.0)	0.788
Malignancy ^‡^	4 (10.5)	6 (14.3)	4 (8.9)	8 (16.0)	0.718
Lab data					
Hb (g/dL) **	10.6 ± 1.2	9.9 ± 0.9	10.3 ± 1.0	10.2 ± 1.7	0.165
Platelet (×1000/μL) **	178.4 ± 56.4	206.0 ± 55.9	173.6 ± 54.2	174.6 ± 54.3	0.021
Fasting sugar (mg/dL) **	114.9 ± 61.4	109.5 ± 42.7	117.9 ± 63.4	113.8 ± 43.2	0.910
Cholesterol (mg/dL) **	163.4 ± 42.2	174.7 ± 40.8	149.1 ± 31.8	151.2 ± 34.2	0.005
Triglyceride (mg/dL) **	153.5 ± 177.5	163.3 ± 108.7	130.4 ± 90.8	112.2 ± 67.3	0.144
Albumin (gm/dL) **	4.0 ± 0.3	3.8 ± 0.4	3.9 ± 0.3	3.9 ± 0.3	0.116
A.S.T. [GOT] (IU/L) **	17.0 ± 4.7	15.8 ± 5.4	16.2 ± 6.8	17.3 ± 5.5	0.579
Alkaline-P (IU/L) **	67.6 ± 19.5	69.3 ± 28.0	82.7 ± 42.9	80.3 ± 32.1	0.077
Ferritin (ng/mL) **	582.4 ± 168.3	569.0 ± 237.0	539.3 ± 416.1	522.6 ± 240.3	0.762
TSAT (%) **	29.8 ± 8.8	32.9 ± 12.4	30.8 ± 13.6	32.4 ± 15.4	0.693
Al (ng/mL) **	6.6 ± 3.9	6.4 ± 2.3	5.5 ± 2.4	7.1 ± 3.7	0.117
Uric acid (mg/dL) **	5.8 ± 1.9	6.5 ± 1.6	6.2 ± 1.7	5.9 ± 1.5	0.175
Na (meq/L) **	137.6 ± 2.7	138.5 ± 3.1	138.4 ± 2.6	137.8 ± 3.2	0.517
K (meq/L) *	4.5 ± 0.6	4.7 ± 0.6	4.5 ± 0.6	4.7 ± 0.7	0.189
iCa (mg/dL) **	4.6 ± 0.4	4.6 ± 0.5	4.6 ± 0.4	4.5 ± 0.5	0.574
P (mg/dL) **	4.9 ± 1.2	5.2 ± 1.2	5.0 ± 1.2	5.4 ± 1.4	0.254
PTH (pg/mL) **	240.0 ± 287.8	247.8 ± 254.2	352.8 ± 356.9	328.2 ± 262.3	0.198
Dialysis parameter					
Single pool Kt/V **	1.4 ± 0.2	1.4 ± 0.2	1.3 ± 0.2	1.4 ± 0.2	0.101
Medication (%)					
Anti-HTN drugs					
ACEI/ARB ^†^	21 (55.3)	26 (61.9)	25 (55.6)	31 (62.0)	0.857
*B*-blockers ^†^	21 (55.3)	27 (64.3)	20 (44.4)	30 (60.0)	0.268
CCB ^†^	27 (71.1)	29 (69.0)	26 (57.8)	33 (66.0)	0.583
Diuretics ^†^	10 (26.3)	7 (16.7)	15 (33.3)	8 (16.0)	0.151
Statins ^†^	13 (34.2)	12 (28.6)	21 (46.7)	20 (40.0)	0.342
Anti-diabetic drugs					
OAD ^†^	14 (36.8)	13 (31.0)	15 (33.3)	14 (28.0)	0.841
Insulin and analogs ^‡^	7 (18.4)	1 (2.4)	12 (26.7)	9 (18.0)	0.022
Statins and fenofibrate ^†^	13 (34.2)	12 (28.6)	21 (46.7)	20 (40.0)	0.342
Antiplatelet ^†^	16 (42.1)	14 (33.3)	25 (55.6)	22 (44.0)	0.218
Calcitriol ^†^	15 (39.5)	11 (26.2)	22 (48.9)	23 (46.0)	0.137
Phosphate binder					
Calcium-based ^†^	21 (55.3)	25 (59.5)	28 (62.2)	29 (58.0)	0.933
Non-calcium-based ^†^	7 (18.4)	11 (26.2)	7 (15.6)	13 (26.0)	0.516

Data are expressed as n (%) for categorical data and as mean ± standard deviation for continuous data.* One-way ANOVA; ** Kruskal–Wallis test; ^†^ Chi-square test; ^‡^ Fisher’s exact test.

**Table 2:** Echocardiographic findings for patients with end-stage kidney disease by LVH type and IVCD.

	Eccentric LVH, Small IVCD (n = 38)	Concentric LVH, Small IVCD (n = 42)	Eccentric LVH, Large IVCD (n = 45)	Concentric LVH, Large IVCD (n = 50)	*p*
Aortic root (mm) **	32.1 ± 4.4	32.4 ± 4.9	32.5 ± 3.9	32.3 ± 4.0	0.971
IVS (mm) **	11.2 ± 1.7	12.8 ± 3.4	12.4 ± 2.1	13.6 ± 2.2	<0.001
LA diameter (mm) **	42.7 ± 5.7	41.0 ± 7.5	46.1 ± 7.1	46.4 ± 8.4	0.001
LVEDD (mm) **	53.6 ± 5.3	47.4 ± 5.3	56.4 ± 6.2	48.8 ± 6.7	<0.001
LVESD (mm) **	32.9 ± 6.1	28.4 ± 5.3	37.1 ± 8.7	30.8 ± 7.3	<0.001
LVPW (mm) **	9.6 ± 1.4	12.4 ± 2.4	10.3 ± 1.1	12.9 ± 1.6	<0.001
LVMI **	139.8 ± 23.4	150.4 ± 50.8	161.5 ± 39.3	164.7 ± 42.7	0.022
RWT (mm) **	0.4 ± 0.0	0.5 ± 0.1	0.4 ± 0.0	0.5 ± 0.1	<0.001
IVC diameter (cm) **	1.2 ± 0.2	1.1 ± 0.2	1.9 ± 0.3	1.9 ± 0.3	<0.001

Data expressed as mean ± standard deviation for continuous data. LVH, left ventricular hypertrophy; IVS, interventricular septum; LA, left atrium; LVEDD, left ventricular end-diastolic diameter; LVESD, left ventricular end-systolic diameter; LVPW, left ventricular posterior wall; LVMI, left ventricular mass index; RWT, relative wall thickness; IVCD, inferior vena cava diameter ** One-way analysis of variance.

**Table 3:** Cox proportional hazard analysis of outcome events in the four groups defined by left ventricular hypertrophy (LVH) and inferior vena cava diameter.

Events	Crude		Model 1		Model 2	
	HR (95% CI)	*p*	HR (95% CI)	*p*	HR (95% CI)	*p*
**MACE**						
Eccentric LVH, small IVCD	Reference	-	Reference	-	Reference	-
Concentric LVH, small IVCD	0.91 (0.34–2.44)	0.851	0.95 (0.35–2.56)	0.921	1.55 (0.50–4.86)	0.449
Eccentric LVH, large IVCD	3.13 (1.41–6.97)	0.005	3.34 (1.43–7.81)	0.005	4.40 (1.58–12.23)	0.004
Concentric LVH, large IVCD	2.30 (1.01–5.21)	0.047	2.72 (1.13–6.53)	0.025	3.60 (1.28–10.12)	0.015
**CV mortality**						
Eccentric LVH, small IVCD	Reference	-	Reference	-	Reference	-
Concentric LVH, small IVCD	7.70 (0.99–60.05)	0.052	8.34 (1.05–66.29)	0.045	8.87 (1.01–77.87)	0.049
Eccentric LVH, large IVCD	10.26 (1.35–77.89)	0.024	10.79 (1.51–77.19)	0.018	10.23 (1.41–74.33)	0.022
Concentric LVH, large IVCD	11.56 (1.56–85.91)	0.017	15.44 (2.15–111.0)	0.007	14.34 (1.99–103.35)	0.008
**Mortality**						
Eccentric LVH, small IVCD	Reference	-	Reference	-	Reference	-
Concentric LVH, small IVCD	1.53 (0.51–4.63)	0.447	1.64 (0.54–4.97)	0.383	2.01 (0.54–7.39)	0.295
Eccentric LVH, large IVCD	2.43 (0.87–6.83)	0.092	2.75 (1.01–7.47)	0.048	3.41 (1.09–10.65)	0.035
Concentric LVH, large IVCD	2.65 (0.98–7.17)	0.055	3.79 (1.41–10.23)	0.008	4.38 (1.44–13.37)	0.009

Model 1: adjusted for age and sex; Model 2: adjusted for age, sex, and all significant variables listed in Table 1 (coronary artery disease, peripheral artery disease, cholesterol level, platelet count, and insulin level).

## Data Availability

The data presented in this study are available on request from the corresponding author.

## References

[1] Kovesdy C.-P. (2022). Epidemiology of chronic kidney disease: An update 2022. Kidney Int. Suppl..

[2] Cozzolino M., Mangano M., Stucchi A., Ciceri P., Conte F., Galassi A. (2018). Cardiovascular disease in dialysis patients. Nephrol. Dial. Transplant..

[3] Cozzolino M., Galassi A., Pivari F., Ciceri P., Conte F. (2017). The Cardiovascular burden in end-stage renal disease. Contrib. Nephrol..

[4] Major R.-W., Cheng M.-R.-I., Grant R.A., Shantikumar S., Xu G., Oozeerally I., Brunskill N.-J., Gray L.-J. (2018). Cardiovascular disease risk factors in chronic kidney disease: A systematic review and meta-analysis. PLoS ONE.

[5] Allon M. (2013). Evidence-based cardiology in hemodialysis patients. J. Am. Soc. Nephrol..

[6] Lavie C.-J., Patel D.-A., Milani R.-V., Ventura H.-O., Shah S., Gilliland Y. (2014). Impact of echocardiographic left ventricular geometry on clinical prognosis. Prog. Cardiovasc. Dis..

[7] Di Lullo L., Gorini A., Russo D., Santoboni A., Ronco C. (2015). Left Ventricular Hypertrophy in Chronic Kidney Disease Patients: From Pathophysiology to Treatment. Cardiorenal Med..

[8] Sayin B.-Y., Oto A. (2022). Left Ventricular Hypertrophy: Etiology-Based Therapeutic Options. Cardiol. Ther..

[9] Bornstein A.-B., Rao S.-S., Marwaha K. (2023). Left ventricular hypertrophy. Progress in Cardiovascular Diseases.

[10] Toprak A., Koc M., Tezcan H., Ozener I.-C., Akoglu E., Oktay A. (2003). Inferior vena cava diameter determines left ventricular geometry in continuous ambulatory peritoneal dialysis patients: An echocardiographic study. Nephrol. Dial. Transplant..

[11] Lee S.-L., Daimon M., Kawata T., Kohro T., Kimura K., Nakao T., Koide D., Watanabe M., Yamazaki T., Komuro I. (2014). Estimation of right atrial pressure on inferior vena cava ultrasound in Asian patients. Circ. J..

[12] Canaud B., Chazot C., Koomans J., Collins A. (2019). Fluid and hemodynamic management in hemodialysis patients: Challenges and opportunities. J. Bras. Nefrol..

[13] Loutradis C., Sarafidis P.-A., Ferro C.-J., Zoccali C. (2020). Volume overload in hemodialysis: Diagnosis, cardiovascular consequences, and management. Nephrol. Dial. Transplant..

[14] Jobs A., Brünjes K., Katalinic A., Babaev V., Desch S., Reppel M., Thiele H. (2017). Inferior vena cava diameter in acute decompensated heart failure as predictor of all-cause mortality. Heart Vessel..

[15] Wu C.-K., Yar N., Kao Z.-K., Chuang M.-T., Chang T.-H. (2022). High Inferior Vena Cava Diameter with High Left Ventricular End Systolic Diameter as a Risk Factor for Major Adverse Cardiovascular Events, Cardiovascular and Overall Mortality among Chronic Hemodialysis Patients. J. Clin. Med..

[16] Lang R.-M., Badano L.-P., Mor-Avi V., Afilalo J., Armstrong A., Ernande L., Flachskampf F.-A., Foster E., Goldstein S.-A., Kuznetsova T. (2016). Recommendations for Cardiac Chamber Quantification by Echocardiography in Adults: An Update from the American Society of Echocardiography and the European Association of Cardiovascular Imaging. Eur. Heart. J. Cardiovasc. Imaging.

[17] Patil S., Jadhav S., Shetty N., Kharge J., Puttegowda B., Ramalingam R., Cholenahally M.-N. (2016). Assessment of inferior vena cava diameter by echocardiography in normal Indian population: A prospective observational study. Indian Heart. J..

[18] Franco A.-C.-C., Carneiro L.-D.-S., Franco R.-S.-M., Góes A.-M.-O. (2022). Influence of sex and age on inferior vena cava diameter and implications for the implantation of vena cava filters. J. Vasc. Bras..

[19] Yilmaz Z., Yildirim Y., Oto F., Aydin F.-Y., Aydin E., Kadiroglu A.-K., Yilmaz M.-E. (2014). Evaluation of volume overload by bioelectrical impedance analysis, NT-proBNP and inferior vena cava diameter in patients with stage 3&4 and 5 chronic kidney disease. Ren. Fail..

[20] Hung S.-C., Kuo K.-L., Peng C.-H., Wu C.-H., Lien Y.-C., Wang Y.-C., Tarng D.-C. (2014). Volume overload correlates with cardiovascular risk factors in patients with chronic kidney disease. Kidney Int..

[21] Blankfield R.-P. (2011). Calculated effect of fluid retention upon velocity of blood flow and turbulence: Implications for atherosclerosis. Clin. Hemorheol. Microcirc..

[22] Maimaitiaili R., Teliewubai J., Zhao S., Tang J., Chi C., Zhang Y., Xu Y. (2020). Relationship Between Vascular Aging and Left Ventricular Concentric Geometry in Community-Dwelling Elderly: The Northern Shanghai Study. Clin. Interv. Aging.

[23] Tsai Y.-C., Tsai H.-J., Lee C.-S., Chiu Y.-W., Kuo H.-T., Lee S.-C., Chen T.-H., Kuo M.-C. (2018). The interaction between N-terminal pro-brain natriuretic peptide and fluid status in adverse clinical outcomes of late stages of chronic kidney disease. PLoS ONE.

[24] Lee M.-J., Doh F.-M., Kim C.-H., Koo H.-M., Oh H.-J., Park J.-T., Han S.-H., Yoo T.-H., Kim Y.-L., Kim Y.-S. (2014). Interdialytic weight gain and cardiovascular outcome in incident hemodialysis patients. Am. J. Nephrol..

[25] Ha E.-T., Cohen M., Peterson S.-J., Aronow W.-S. (2021). Eccentric hypertrophy predicts adverse events in patients undergoing percutaneous coronary intervention for acute coronary syndrome. Arch. Med. Sci. Atheroscler. Dis..

[26] Tsai Y.-C., Chiu Y.-W., Tsai J.-C., Kuo H.-T., Hung C.-C., Hwang S.-J., Chen T.-H., Kuo M.-C., Chen H.-C. (2015). Association of Fluid Overload with Cardiovascular Morbidity and All-Cause Mortality in Stages 4 and 5 CKD. Clin. J. Am. Soc. Nephrol..

[27] Onofriescu M., Siriopol D., Voroneanu L., Hogas S., Nistor I., Apetrii M., Florea L., Veisa G., Mititiuc I., Kanbay M. (2015). Overhydration, Cardiac Function and Survival in Hemodialysis Patients. PLoS ONE.

[28] Schwermer K., Hoppe K., Kałużna M., Dopierała M., Olszewska M., Nealis J., Łukawiecka A., Oko A., Pawlaczyk K. (2021). Overhydration as a modifiable cardiovascular risk factor in patients undergoing hemodialysis. Pol. Arch. Intern. Med..

[29] Chao C.-T., Liao M.-T., Wu C.-K. (2023). Left Ventricular Hypertrophy Geometry and Vascular Calcification Co-Modify the Risk of Cardiovascular Mortality in Patients with End-Stage Kidney Disease: A Retrospective Cohort Study. J. Atheroscler. Thromb..

[30] Koren M.-J., Devereux R.-B., Casale P.-N., Savage D.-D., Laragh J.-H. (1991). Relation of left ventricular mass and geometry to morbidity and mortality in uncomplicated essential hypertension. Ann. Intern. Med..

[31] Muiesan M.-L., Salvetti M., Monteduro C., Bonzi B., Paini A., Viola S., Poisa P., Rizzoni D., Castellano M., Agabiti-Rosei E. (2004). Left ventricular concentric geometry during treatment adversely affects cardiovascular prognosis in hypertensive patients. Hypertension.

[32] Lieb W., Gona P., Larson M.-G., Aragam J., Zile M.-R., Cheng S., Benjamin E.-J., Vasan R.-S. (2014). The natural history of left ventricular geometry in the community: Clinical correlates and prognostic significance of change in LV geometric pattern. JACC Cardiovasc. Imaging.

[33] Cioffi G., Viapiana O., Orsolini G., Ognibeni Sonographer F., Dalbeni A., Gatti D., Adami G., Fassio A., Rossini M., Giollo A. (2021). Left ventricular hypertrophy predicts poorer cardiovascular outcome in normotensive normoglycemic patients with rheumatoid arthritis. Int. J. Rheum. Dis..

[34] Le T.-T., Lim V., Ibrahim R., Teo M.T., Bryant J., Ang B., Su B., Aw T.-C., Lee C.-H., Bax J. (2021). The remodelling index risk stratifies patients with hypertensive left ventricular hypertrophy. Eur. Heart. J. Cardiovasc. Imaging.

[35] de Roij van Zuijdewijn C.-L., Hansildaar R., Bots M.-L., Blankestijn P.-J., van den Dorpel M.-A., Grooteman M.-P., Kamp O., ter Wee P.-M., Nubé M.-J. (2015). Eccentric Left Ventricular Hypertrophy and Sudden Death in Patients with End-Stage Kidney Disease. Am. J. Nephrol..

